# Pre-Clinical Proof of Concept: Intra-Carotid Injection of Autologous CD34-Positive Cells for Chronic Ischemic Stroke

**DOI:** 10.3389/fmed.2022.681316

**Published:** 2022-03-11

**Authors:** Yuko Ogawa, Yuka Okinaka, Akie Kikuchi-Taura, Orie Saino, Ayumi Tani-Yokoyama, Satoru Masuda, Miki Komatsu-Horii, Yoshihiko Ikemoto, Atsuhiko Kawamoto, Masanori Fukushima, Akihiko Taguchi

**Affiliations:** ^1^Department of Regenerative Medicine Research, Foundation for Biomedical Research and Innovation at Kobe, Hyogo, Japan; ^2^Translational Research Center for Medical Innovation, Foundation for Biomedical Research and Innovation at Kobe, Hyogo, Japan; ^3^Foundation of Learning Health Society Institute, Nagoya, Japan

**Keywords:** chronic stroke, peripheral blood CD34 positive cells, hematopoietic stem cells, internal carotid artery, pre-clinical proof of concept

## Abstract

This study was conducted to evaluate the safety and efficacy of human peripheral blood CD34 positive (CD34^+^) cells transplanted into a murine chronic stroke model to obtain pre-clinical proof of concept, prior to clinical testing. Granulocyte colony stimulating factor (G-CSF) mobilized human CD34^+^ cells [1 × 10^4^ cells in 50 μl phosphate-buffered saline (PBS)] were intravenously (iv) or intra-carotid arterially (ia) transplanted 4 weeks after the induction of stroke (chronic stage), and neurological function was evaluated. In this study, severe combined immune deficiency (SCID) mice were used to prevent excessive immune response after cell therapy. Two weeks post cell therapy, the ia CD34^+^ cells group demonstrated a significant improvement in neurological functions compared to the PBS control. The therapeutic effect was maintained 8 weeks after the treatment. Even after a single administration, ia transplantation of CD34^+^ cells had a significant therapeutic effect on chronic stroke. Based on the result of this pre-clinical proof of concept study, a future clinical trial of autologous peripheral blood CD34^+^ cells administration in the intra-carotid artery for chronic stroke patients is planned.

## Introduction

Stroke is characterized by severe sequelae and a significant reduction in patient quality-of-life. With the recent development of stem cell research, cell therapy for stroke has been studied as a new therapeutic strategy (1–5). We have shown that hematopoietic stem cells (HSCs) induce the regeneration of cerebral nerves along with vascular regeneration in a mouse model of middle cerebral artery occlusion (MCAO) (6). In addition, we conducted a study on HSC transplantation for sub-acute stroke patients (7) and found a therapeutic effect. Furthermore, we have shown that the mechanism of action of HSCs is the provision of energy to damaged vascular endothelial cells (8). However, most cell therapies target the acute or subacute phase, with few therapies for chronic stroke. Nervous system function is almost completely lost when an infarction transitions from the acute to chronic stroke phase. Therefore, it has been argued that stem cells cannot have a therapeutic effect at the chronic stage. However, in our previous study, we demonstrated that combination therapy of training and bone-marrow-derived HSCs had significant therapeutic effects in a murine chronic stroke model (9). Since it has been suggested that it may also have a therapeutic effect in humans, we plan to conduct a human clinical trial of HSC therapy for chronic stroke using G-CSF mobilized autologous peripheral blood CD34 positive (CD34^+^) cells. The administration of autologous peripheral blood CD34^+^ cells has already been performed safely in many clinical trials, in which the safety of the cell therapy has been reported (10–14).

Our study aims to evaluate the safety and efficacy of transplanted human peripheral blood CD34^+^ cells using a murine chronic stroke model in order to obtain pre-clinical proof of concept for our planned clinical trial. Furthermore, optimal cell number and the route of administration will be determined.

## Materials and Methods

The following study was approved by the Animal Care and Use Committee of the Institute of Biomedical Research and Innovation (Kobe, Hyogo, Japan), and complies with the Guide for the Care and Use of Animals published by the Ministry of Education, Culture, Sports, Science, and Technology in Japan.

### Animals

All animal experiments were approved by the Animal Care and Use Committee of the Foundation for Biomedical Research and Innovation (date of approval; November 5th, 2019, No. 18-09) and complied with the Guide for the Care and Use of Animals published by the Japanese Ministry of Education, Culture, Sports, Science, and Technology. This research was in line with current recommendations in the field for translational cell therapy research in stroke (15).

### Stroke Model

A murine stroke model with excellent reproducibility was applied in 5-weeks-old male severe combined immune deficiency (SCID) mice (C.B-17/lcr-*scid*/*scid*Jcl: Oriental Yeast, Tokyo, Japan), as we previously described (6). To prevent excessive immune response after cell therapy, SCID mice were used. Briefly, permanent focal cerebral ischemia was induced by permanent ligation and disconnection of the distal portion of the left middle cerebral artery (MCA) using bipolar forceps under isoflurane inhalation anesthesia (3% for induction and 2% for maintenance). During surgery, rectal temperature was monitored and controlled at 37.0 ± 0.2°C by a feedback-regulated heating pad. After the MCAO insult, mice were kept in a 37°C incubator until the anesthetic wore off to prevent a drop in body temperature. The survival rate of this model was approximately 100% for 90 days post MCAO insult, and the load on mice during and after the creation of the cerebral infarction was relatively small. Cerebral blood flow (CBF) in the MCA area was also monitored. Mice showing a ≥75% decrease in CBF immediately after MCAO were used experimentally (100% success rate). All prepared mice underwent the surgery successfully. In this study, cell therapy and behavioral tests were performed in a randomized, blinded fashion. Once completed, data was unblinded and analyzed.

### Isolation of CD34^+^ Cells From Human Peripheral Blood

Human peripheral blood CD34^+^ cells were isolated from G-CSF mobilized apheresis products (Stem express, Folsom, USA). CD34^+^ cells were isolated by using CliniMACS® (Miltenyi Biotec B.V. & Co. KG, Bergisch Gladbach, Germany). The CD34^+^ cells fraction had a purity of 95.7% and total cell viability of 82.92%, as determined by flow cytometry analysis using a CD34-specific monoclonal antibody (IM3630a; Beckman Coulter, Marseille, France).

### Cell Administration

CD34^+^ cells were diluted to an appropriate concentration with phosphate-buffered saline (PBS), and immediately used for our experiments. At 28 days after the MCAO insult, 1 × 10^4^ CD34^+^ cells in 50 μl PBS were injected into the tail vein (iv) or the carotid artery (ia). The left common carotid artery (CCA) was used for intra-arterial injections. Briefly, a longitudinal skin incision was made at the center of the cervix, and the left CCA was isolated under isoflurane inhalation anesthesia. After clipping the proximal portion of the CCA, cells were infused through a 35G needle. Subsequently, the hole was infilled with surgical glue (Aron Alpha A; Sankyo, Tokyo, Japan), and the clip was released (16). Control PBS (50 μl) was injected into the tail vein of SCID mice (Control PBS, *n* = 8; CD34^+^ iv, *n* = 8; CD34^+^ ia, *n* = 9).

### Behavioral Testing

**Figure 1:**
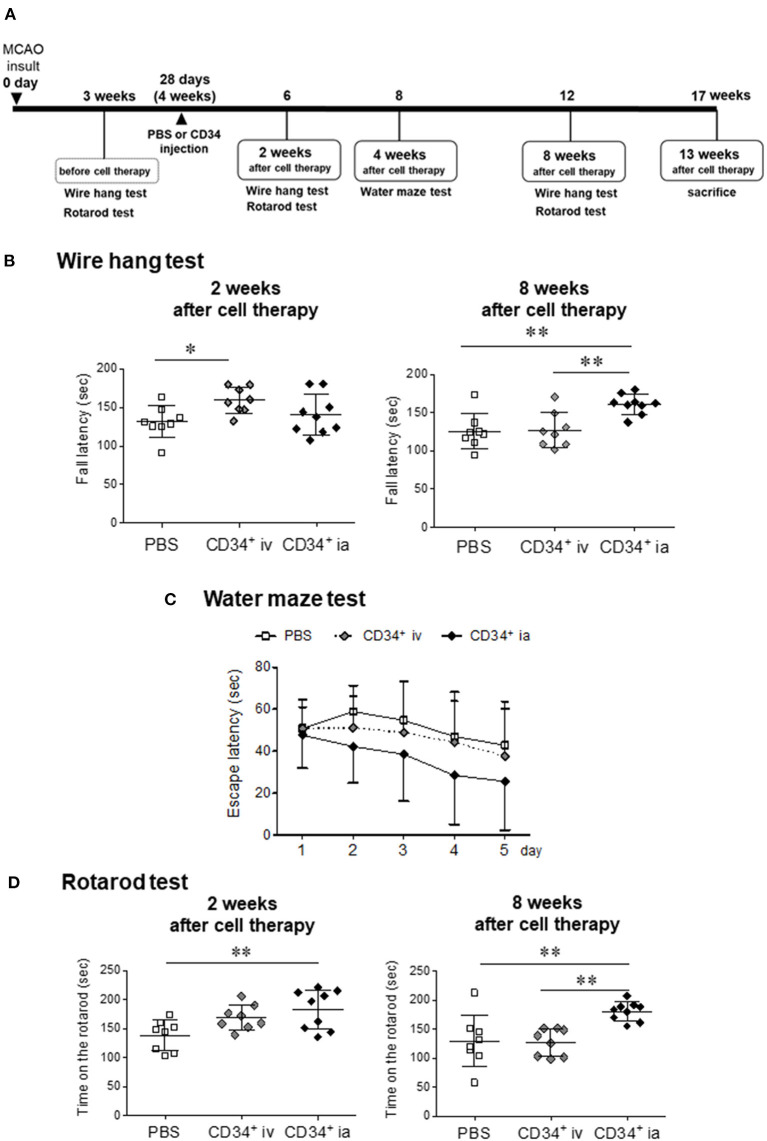
Effect of CD34 transplantation for chronic stroke. **(A)** Experimental schedule in this study. **(B)** The neuromuscular function was measured by the wire hang test before treatment, and 2 and 8 weeks after cell therapy. The latency to fall was measured. The hanging time of CD34^+^ ia treatment groups at 8 weeks after treatment showed a significant longer level compared with the PBS and CD34^+^ iv treatment group. **(C)** Water maze test was performed at 4 weeks after the treatment. We measured the time until mice reached onto a platform submerged in a pool for five consecutive days. We presented the average of the 5-day-session data. The escape latency over 5 days of training tended to decrease in CD34^+^ ia-treated mice, but there was no significant difference between all groups because of large individual differences in each group. **(D)** Sensorimotor skills were evaluated in the rotarod test before treatment, and 2 and 8 weeks after cell therapy. The rotarod performance means time on the rotarod. Significant difference was observed between PBS and CD34^+^ ia treatment groups at 2 weeks after the cell treatment. The therapeutic effect persisted 8 weeks after the treatment. MCAO, permanent middle cerebral artery occlusion; CD34^+^, autologous peripheral blood CD34 positive cells, iv; intravenous administration group; ia, intra-arterial administration group. ^*^p < 0.05, ^**^p < 0.01.

The experimental design is shown in Figure 1A. To assess motor function, mice were subjected to behavioral testing using the wire hang, water maze, and rotarod tests. All behavioral tests were conducted at the optimal time, determined by a preliminary study (9). Prior to administration of cell therapy, behavior tests were conducted to confirm that there were no significant differences in behavioral disorders among individuals. Based on the validation results in previous studies, the correct behavioral assessment was conducted at the right time.

#### Wire Hang Test

The wire hang test seeks to evaluate muscular strength or motor function. In this test, each mouse was placed on a wire mesh plate and allowed to accommodate to this environment for 5 s. The wire mesh plate was then gently inverted and secured to the top of a cubic open-topped glass box (25 cm × 25 cm × 25 cm). Latency to fall was measured with a maximum trial time of 3 min. This trial was repeated five times with an interval of 1 min.

#### Water Maze Test

To evaluate learning and memory function, the water maze test was performed. A circular swimming pool (diameter, 100 cm; depth, 40 cm) was placed in a test room and filled with water. A circular platform (diameter, 8 cm) was submerged 1 cm below the water surface in the center of one quadrant of the pool. In order to train the mice to escape, each mouse was subjected to 5 training trials per day for 5 consecutive days. In each training trial, the mouse was released into the water with its head facing the outer edge of the pool, and the releasing place was selected, except the one where the platform was hidden. The training trial terminated when the mouse reached the platform and remained on it for 10 s. If the platform was not found within 60 s, the mouse was guided to the platform by the experimenter and kept there for 3 s. The order of the release points was varied on a daily basis, with pseudorandom sequences for each mouse. On the next day of the last training day, all mice were subjected to a probe test trial, in which the platform was removed. Each mouse was released into the south quadrant of the pool and was allowed to swim freely for 60 s. All trials were recorded using video analysis systems (Etho Vision XT5; Noldus, Wageningen, Netherlands).

#### Rotarod Test

Sensorimotor skills were evaluated in the rotarod test. A rotarod drum was accelerated from 4 to 40 rpm over 5 min (Muromachi Kikai Co. Ltd., Tokyo, Japan). Each mouse was placed on the stationary drum. Five seconds later, the rotor was started. The time until the mouse fell off the rotating drum was recorded. This trial was repeated five times with an interval of 1 min. The average time for fall-offs from the drum was used for statistical analysis.

### Data Analysis

Statistical analyses were run on Prism 9 (GraphPad Software, San Diego, CA, USA). Results of wire hang test and rotarod test were assessed using one-way analysis of variance (ANOVA), followed by *post-hoc* analysis using Dunnett's test. Result of the repeated evaluation, water maze test, was assessed using two-way ANOVA, followed by the Bonferroni correction for repeated measure. Differences were considered statistically significant at *p* < 0.05. All results were expressed as the mean ± standard deviation (SD).

## Results

### Mortality

In this study, 25 mice survived for 13 weeks after cell transplantation, with no dropout. No safety concerns were observed during pathological autopsy. Both ia and iv administration of CD34^+^ cells were deemed safe in the murine chronic stroke model.

### Wire Hang Test

To evaluate neuromuscular function at the chronic stroke, we used the wire hang test before the treatment and at 2 and 8 weeks after treatment (Figure 1B). Before the treatment, the hanging time of all groups showed no difference (data not shown). Two weeks after treatment, the hanging time was significantly longer in CD34^+^ cells iv-treated group compared with PBS group. Eight weeks after the treatment, the therapeutic effect of CD34^+^ cells iv-treated group was attenuated and became same level as PBS group. On the other hand, therapeutic effect of CD34^+^ cells ia-treated group was not observed 2 weeks after the cell therapy. However, after 8 weeks, the hanging time was significantly longer in CD34^+^ cells ia group compared with the PBS and CD34^+^ cells iv-treated groups.

### Water Maze Test

To investigate the spatial learning ability and escape reaction, mice were subjected to water maze test at 4 weeks after cell therapy (Figure 1C). At day 1 trial, there was no difference between all groups. CD34^+^ cells ia-treated group showed rapid decrease in the escape latency over 5 days of training, but there was no significant intergroup difference because of large individual differences in each group. These results indicated that escape reaction and spatial learning ability tended to be improved by CD34^+^ cells ia transplantation with repeated training.

### Rotarod Test

Sensorimotor skills or endurance were evaluated at 2 and 8 weeks after cell therapy (Figure 1D). At 2 weeks after cell therapy, the hanging time of CD34^+^ cells iv-treated group was found to be longer than that of the PBS group, but there was no significant difference. This therapeutic effect of CD34^+^ cells iv-treated group disappeared 8 weeks after the cell therapy. On the other hand, CD34^+^ cells ia-treated group showed a significant difference compared to the PBS group at 2 weeks after cell therapy, and the therapeutic effect had persisted 8 weeks after the treatment.

### Adverse Events After Cell Therapy

After the behavioral evaluations were completed, pathological autopsy was performed at 13 weeks after the cell therapy. No abnormal findings such as oncogenesis or inflammations were observed visually in the lungs, liver, digestive organs, spleen, kidneys, and reproductive organs (data not shown).

## Discussion

Intra-carotid artery injection of G-CSF mobilized human CD34^+^ cells significantly improves neurological functions in a murine chronic stroke model, without any adverse events. In this study, we obtained pre-clinical proof of concept for the ia transplantation of CD34^+^ cells for chronic stroke. This is the first report to demonstrate a significant therapeutic effect and safety of ia transplantation of CD34^+^ cells for the chronic phase of stroke.

Few clinical trials in which HSCs were ia administered in the chronic phase of stroke have been reported (17–19). These reports indicated that HSCs could restore a certain level of nerve function without adverse events. In this study, the dose and route of administration of CD34^+^ cells for this study were determined based on the results of our previous pre-clinical studies and clinical findings. iv administration of CD34^+^ cells (1 × 10^5^ cells/mouse) after sub-acute stroke enhanced neurogenesis *via* angiogenesis in a mouse model (6). In a study using human bone-marrow-derived CD133^+^ HSCs, the brain protective effect in the ia transplantation group was equivalent to that in the iv administration group, which received ten times more cells (16). There is a limited number of CD34^+^ cells that can be collected from human peripheral blood. Therefore, from experience, 5 × 10^5^ cells/kg is the cell number upper limit of which can be obtained from elderly blood samples and hence, the human clinical study dose has been set at 5 × 10^5^ cells/kg. In this pre-clinical experiment, the weight of each mouse was calculated as 20 g, and 1 × 10^4^ cells were used per mouse. From the results of this study, it was clear that the ia transplantation of CD34^+^ cells (1 × 10^4^ cells/mouse, single dose) was a more successful and effective treatment compared with iv transplantation. In terms of the therapeutic effect of HSCs used in the treatment of chronic stroke, ia administration and 5 × 10^5^ cells/kg were the optimal route and dose, respectively.

Several studies have reported the occurrence of micro-strokes after ia administration of cells (20, 21). However, these studies used bone marrow mesenchymal stem cells (BM-MSCs), which are adhesion cells. We believe cell characters of non-adherent circulating blood cells (CD34^+^ cells) are very different from adherent cells. In general, BM-MSCs are large (cell diameter: 17.2 ± 1.9 μm) and tend to form emboli after administration (capillary diameter: ~5–10 μm). Therefore, it is expected that iv/ia injection of large adhesive cells cause cell embolism in blood vessels. In contrast, the HSCs used in our study are non-adherent, smaller (diameter: ~4.6 ± 0.2 μm) with a low risk of embolisation. In this study, we administered purified cells present in autologous peripheral blood. We have shown that ia injection of CD133^+^ cells (both CD34 and CD133 are HSC markers and the majority of CD133^+^ cells are CD34^+^) suppressed microvasculature shrinkage in ischemic brain (16). Clinical trials have been conducted for diseases other than cerebral infarction, while no adverse events have been reported, and the safety of this cell therapy is considered to be high. In addition, it has been shown that clot-derived contaminants in transplanted cells impaired the therapeutic effect of HSCs (22). The cells administered in this study were purified CD34^+^ cells, and it was likely that the absence of blood clots led to a strong therapeutic effect. In terms of the therapeutic mechanisms for CD34^+^ cells, it is possible that the administered cells differentiated into vascular endothelial cells of the brain and promoted angiogenesis (10, 23). It has also been suggested that HSCs supply energy sources, including glucose, to damaged endothelial cells *via* cell-cell interactions through gap junctions (8). The site of action of HSCs are cerebrovascular endothelial cells. The number of cells that reach the brain differs between iv and ia administration. Therefore, it was thought that ia administration led to an enhanced therapeutic effect as more cells reached the brain. However, iv administration of cells had a weaker effect; it was thought that this might have led to the loss of therapeutic effect at week eight because the therapeutic effect could not maintain its activity, even though it caused functional improvement after 2 weeks.

The results of this study indicated that ia administration of CD34^+^ cells had a significant therapeutic effect on chronic cerebral infarction, even after a single administration. Based on this pre-clinical proof of concept, we will conduct a clinical trial of autologous peripheral blood CD34^+^cell administration in the internal carotid artery for patients with chronic stroke. The main outcome evaluation will be the Fugl–Meyer assessment (FMA) of upper limb function as the motor function of chronic stroke patients is expected to improve after cell therapy.

In conclusion, CD34^+^ cell transplantation is likely to improve neurological prognoses, even in the chronic phase, based on pre-clinical studies using a murine chronic stroke model. If this treatment is established as the standard treatment for chronic stroke, the benefits to the healthcare economy will be high.

## Data Availability Statement

The original contributions presented in the study are included in the article/supplementary material, further inquiries can be directed to the corresponding author.

## Ethics Statement

The animal study was reviewed and approved by the Animal Care and Use Committee of Foundation for Biomedical Research and Innovation at Kobe.

## Author Contributions

YOg performed the experiments (behavioral test), analyzed the data, and wrote the manuscript. YOk performed the experiments (animal model). AK-T performed cell therapy, and OS analyzed the data. AT-Y and MK-H isolated CD34^+^ cells from human peripheral blood, and SM and YI measured the purity and cell viability by FACS analysis. AK and MF supervised the project and revised the manuscript critically for important intellectual content. AT designed the study and prepared this manuscript. All authors gave their approval to the manuscript.

## Funding

This study was supported by the Japan Agency for Medical Research and Development (AMED) under Grant Number JP19bk0104004h0002 and 20bm0404069h0001, and JSPS KAKENHI/Grant Number 21K09194.

## Conflict of Interest

The authors declare that the research was conducted in the absence of any commercial or financial relationships that could be construed as a potential conflict of interest.

## Publisher's Note

All claims expressed in this article are solely those of the authors and do not necessarily represent those of their affiliated organizations, or those of the publisher, the editors and the reviewers. Any product that may be evaluated in this article, or claim that may be made by its manufacturer, is not guaranteed or endorsed by the publisher.
